# Efficacy and safety of angiogenesis inhibitors in small-cell lung cancer

**DOI:** 10.18632/oncotarget.13588

**Published:** 2016-11-25

**Authors:** Heng Lin, Lina Li, Shuimei Luo, Sijing Zhou, Ruifen Shen, Haitao Yang, Huijuan Chen, Xianhe Xie

**Affiliations:** ^1^ Department of Chemotherapy, The First Affiliated Hospital, Fujian Medical University, Fuzhou, Fujian, China; ^2^ Department of Oncology, Fuzhou Pulmonary Hospital, Fuzhou, Fujian, China

**Keywords:** angiogenesis inhibitors, chemotherapy, targeted therapy, small-cell lung cancer, meta-analysis

## Abstract

**Objective:**

The purpose of this study was to investigate the efficacy and safety of angiogenesis inhibitors for small-cell lung cancer (SCLC).

**Methods:**

Totally, 16 controlled trials (1898 cases) involving angiogenesis inhibitors plus chemotherapy (ACT group) versus chemotherapy alone group (CT group) were identified from PubMed, EMBASE, Cochrane Library and Wanfang Data before March 2016.

**Results:**

Compared with CT group, ACT group obtained a significant benefit on objective response rate (ORR) (RR = 1.34; 95% CI = 1.19-1.51; *P* < 0.00001) and a trend of prolonging progression-free survival (PFS) (HR = 0.86; 95% CI = 0.73-1.01; *P* = 0.07) without improving overall survival (OS) (HR = 1.05; 95% CI = 0.94-1.17; *P =* 0.36). Remarkably, subgroup analysis showed that the antibodies targeting VEGF significantly prolonged PFS (HR = 0.76; 95% CI = 0.64-0.90; *P* = 0.001). With regard to toxicity, there was no significant difference in severe adverse events (AEs, Grade≥3) between two groups except that gastrointestinal symptom, hypertension, metabolic disorders, neurology and pain were higher in ACT group.

**Conclusion:**

Compared with chemotherapy alone, antibodies targeting VEGF plus chemotherapy significantly improved ORR and prolonged PFS with an acceptable toxicity profile for patients with SCLC. Therefore, angiogenesis inhibitors, especially antibodies targeting VEGF, combining with chemotherapy may be a potential promising strategy in managing SCLC.

## INTRODUCTION

Small-cell lung cancer (SCLC) accounts for approximately 15% of all newly diagnosed cases of lung cancer, which is the leading cause of cancer-related death worldwide [1]. About two-thirds of SCLC patients are initially diagnosed at advanced stage, appearing a poor prognosis [2]. For these patients, being unsuitable for surgery or radiotherapy, chemotherapy (CT) (ie, a platinum agent with etoposide or irinotecan) is the main approach [2–5]. Despite the initial high response rate of SCLC to chemotherapy, it can only prolong the median survival time to 7-11 months [6]. Moreover, the first-line therapy has remained essentially unchanged for the past two decades. Therefore, it is urgently needed to seek a multimodality therapy to break the bottleneck of SCLC treatment.

Since preclinical studies demonstrated that angiogenesis played a crucial role in tumor growth [7], it is reasonable to make an attempt to combine angiogenesis inhibitors with chemotherapy. Large randomized controlled trials (RCT) on non-small-cell lung cancer showed the superiority of antiangiogenic agents plus chemotherapy over chemotherapy alone in terms of objective response rate (ORR), overall survival (OS) and progression-free survival (PFS) [8–10]. As for SCLC, some single-arm trials showed favorable efficacy and safety of angiogenesis inhibitors plus chemotherapy [11–13] while other trials failed to confirm that [14, 15]. Thus, the role of angiogenesis inhibitors plus chemotherapy in managing SCLC remains controversial.

Thereby, we performed a meta-analysis to investigate the efficacy and safety of angiogenesis inhibitors plus chemotherapy versus chemotherapy alone.

## RESULTS

### Study selection and characteristics

**Figure 1:**
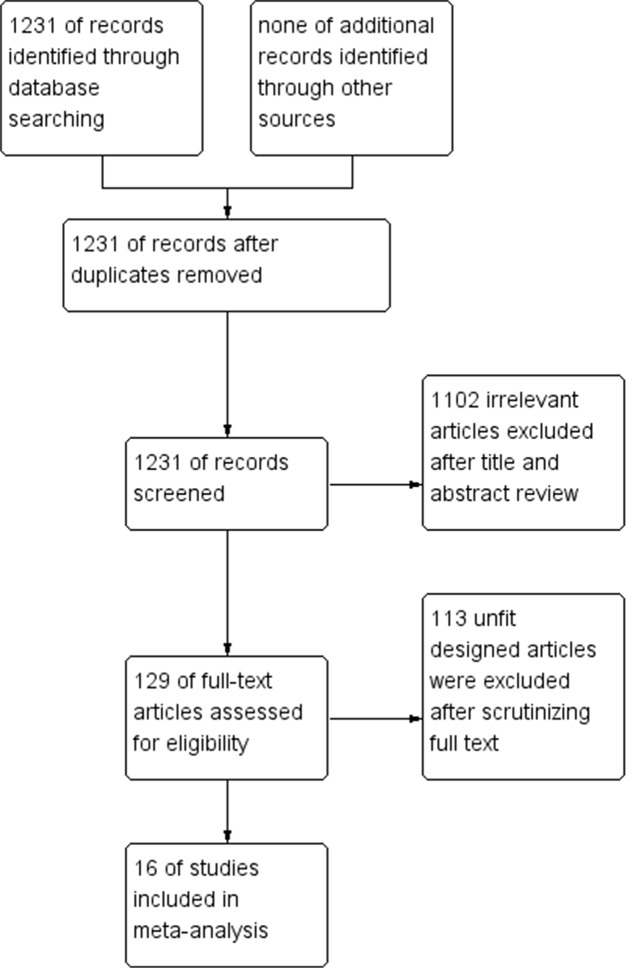
A flow chart on selection included trials in the Meta-analysis

Totally, 1231 articles were screened after searching the relevant databases. By verifying related terms of the study titles and abstracts, 1102 irrelevant articles were removed. Additionally, another 113 unfit designed articles were excluded after scrutinizing full text. Eventually, 16 clinical control trials [16–31] were included. A flowchart depicting inclusion was shown in Figure 1.

There were 1898 patients in the sixteen selected controlled trials, including 956 patients with angiogenesis inhibitors plus chemotherapy and 942 patients with chemotherapy alone. One of these trials involved in ziv-aflibercept plus CT versus CT [16]; two bevacizumab plus CT versus CT [17, 18]; six rh-Endostatin plus CT versus CT [19–24]; six thalidomide plus CT versus CT [25–30]; one vandetanib plus CT versus CT [31]. The populations were comparable with respect to demographics, clinical parameters, stage at initial diagnosis in different clinical settings. These results were summarized in Table 1. Among these 16 trials, two were phase III clinical trials [29, 30]; one phase II- III trial [18]; four phase II trials [16, 17, 19, 31] while nine studies did not mention a trial phase [20–28]. Outcomes included ORR, OS, PFS and severe adverse events (AEs, Grade≥3).

**Table 1:** Baseline characteristic of trials included for analysis

StudyID	Country	Trial phase	Line	N(A/C)	Ages(A/C, Years)	Male(A/C, %)	PS ≤ 2 (A/C, %)	Extensive(A/C, %)	Interventions		Outcomes			
									ACT group	CT group	ORR	OS	PFS	AEs (grade≥3)
Allen et al.2014	America	II	2	97/92	62.0/62.0	44.33/51.09	100.00/100.00	68.04/70.65	Ziv-aflibercept+topotecan	topotecan	Y	Y	Y	Y
Spigel et al.2011	America	II	1	52/50	60/64	50.00/60.00	100.00/100.00	100.00/100.00	Bevacizumab +PE/EC	placebo+PE/EC	Y	Y	Y	Y
Pujol et al.2015	France	II-III	1	37/37	61.2/60.1	67.57/70.27	97.30/100.00	100.00/100.00	Bevacizumab+ PCDE/PE	PCDE/PE	N	Y	Y	Y
Lu et al.2015	China	II	1	69/69	57.7/58.2	81.16/82.61	100.00/100.00	100.00/100.00	rh-Endostatin + EC	EC	Y	Y	Y	Y
Luo et al.2013	China	N	N	19/24	56/57	78.95/79.17	N	N	rh-Endostatin + EC	EC	Y	N	N	Y
Dai et al.2012	China	N	N	50/50	N	N	100.00/100.00	N	rh-Endostatin + PE	PE	Y	N	N	Y
Li et al.2010	China	N	N	24/24	59/56.5	62.50/66.67	100.00/100.00	58.33/54.17	rh-Endostatin + PE	PE	Y	N	N	Y
Wang et al.2011	China	N	N	20/20	56/57.2	60.00/65.00	100.00/100.00	55.00/50.00	rh-Endostatin + PE	PE	Y	N	N	Y
Hu et al.2011	China	N	N	45/44	56.5/57.8	73.33/79.55	100.00/100.00	46.67/43.18	rh-Endostatin + PT	PT	Y	N	N	N
Liu et al.2011	China	N	N	11/11	52.64/55.63	45.45/54.55	100.00/100.00	72.73/63.64	Thalidomide + PE	PE	Y	N	N	Y
Cheng et al.2015	China	N	≥2	28/28	57.9/58.1	67.86/64.29	N	N	Thalidomide + PI	PI	Y	N	N	Y
Liu et al.2015	China	N	N	25/25	52.45/54.63	32.00/24.00	100.00/100.00	44.00/28.00	Thalidomide + PE	PE	N	N	N	N
Liu et al.2013	China	N	2	12/12	N	N	100.00/100.00	N	Thalidomide + PI	PI	Y	N	N	Y
Pujol et al.2007	France	III	1	49/43	59.5/59.6	79.59/79.07	100.00/100.00	100.00/100.00	Thalidomide + PCDE	placebo+PCDE	N	Y	Y	Y
Lee et al.2009	UK	III	1	365/359	65/65	57.81/55.99	96.44/91.92	51.51/46.80	Thalidomide + EC	placebo + EC	N	Y	Y	Y
Arnold et al.2007	Canada	II	mainten-ance	53/54	56.9/62.4	50.94/57.41	100.00/100.00	56.60/57.41	Vandetanib	placebo	N	Y	Y	Y

Abbreviations: N(A/C): number of patients (Angiogenesis inhibitors plus chemotherapy group/Chemotherapy alone group); PS: performance status; ACT: angiogenesis inhibitors plus chemotherapy; CT: chemotherapy; PE: cisplatin and etoposide; EC: etoposide and carboplatin; PCDE: cisplatin - cyclophosphamide - epidoxorubicin – etoposide; PT: cisplatin and topotecan; PI: cisplatin and irinotecan; ORR: objective response rate; OS: overall survival; PFS: progression-free survival; AEs: adverse events; N:no mention in the paper; Y: mentioned in the paper.

Data for all characteristics were summarized in Table 2. Gender, ECOG PS (Eastern Cooperative Oncology Group performance status) and stage were available for 14, 6, 12 of the 16 trials, respectively. The number of withdrawn patients was approximately the same in each trial.

**Table 2:** Characteristics of included patients

	ACT-group (%) N=956 (100%)	CT-group (%) N=942 (100%)
Sex		
Male	534 (56%)	539 (57%)
Female	349 (36%)	327 (35%)
unknown	73 (8%)	76 (8%)
ECOG PS		
0	138 (14%)	177 (19%)
1	416 (44%)	385 (41%)
2	121 (13%)	78 (8%)
3	13 (1%)	29 (3%)
unknown	268 (28%)	273 (29%)
Stage		
Extensive	556 (58%)	519 (55%)
Limited	280 (29%)	295 (31%)
unknown	120 (13%)	128 (14%)

The information about the types of agents, dosage, duration and sequence administrated in different trials was summarized in Table 3.

**Table 3:** Treatment regimens of trials included for analysis

StudyID	Treatment regimens (each 21-day cycle)	
	ACT group	CT group
Allen et al.2014	Ziv-aflibercept 6 mg/kg on day 1+ topotecan 4 mg/m^2^ on days 1, 8 and 15	topotecan 4 mg/m^2^ on days 1, 8 and 15
Spigel et al.2011	Bevacizumab 15 mg/kg on day 1 + etoposide 100 mg/m^2^ on days 1-3 + cisplatin 75 mg/m^2^ or carboplatin AUC = 5 mg/mL/min on day 1	placebo 15 mg/kg on day 1+ etoposide 100 mg/m^2^ on days 1-3 + cisplatin 75 mg/m^2^ or carboplatin AUC = 5 mg/mL/min on day 1
Pujol et al.2015	Bevacizumab 7.5 mg/kg on day 1 + PS 0-1: 4'-epidoxorubicin 30 mg/m^2^; on day 1 + cisplatin 75 mg/m^2^; on day 2+ etoposide 75 mg/m^2^; on days 1-3 + cyclophosphamide 300 mg/m^2^; on days 1-3/ PS 2: cisplatin 80 mg/m^2^; on day 2 + etoposide 120 mg/m^2^; on days 1-3	PS 0-1: 4'-epidoxorubicin 30 mg/m^2^; on day 1 + cisplatin 75 mg/m^2^; on day 2+ etoposide 75 mg/m^2^; on days 1-3 + cyclophosphamide 300 mg/m^2^; on days 1-3/ PS 2: cisplatin 80 mg/m^2^; on day 2 + etoposide 120 mg/m^2^; on days 1-3
Lu et al.2015	rh-Endostatin 7.5 mg/m^2^ on days 1-14 + etoposide 60 mg/m^2^ on days 1-5 + carboplatin AUC = 5 mg/ml/min on day 1	etoposide 60 mg/m^2^ on days 1-5 + carboplatin AUC = 5 mg/ml/min on day 1
Luo et al.2013	rh-Endostatin 15 mg on days 1-14 + etoposide 100 mg on days 1-5 + carboplatin 400-500 mg on day 1	etoposide 100 mg on days 1-5 + carboplatin 400-500 mg on day 1
Dai et al.2012	rh-Endostatin 15 mg on days 1-14 + etoposide 100 mg/m^2^ on days 1-5 + cisplatin 20 mg/m^2^; on days 2-5	etoposide 100 mg/m^2^ on days 1-5 + cisplatin 20 mg/m^2^; on days 2-5
Li et al.2010	rh-Endostatin 15 mg on days 1-14 + etoposide 100 mg/m^2^ on days 1-5 + cisplatin 80 mg/m^2^; on day 2	etoposide 100 mg/m^2^ on days 1-5 + cisplatin 80 mg/m^2^; on day 2
Wang et al.2011	rh-Endostatin 15 mg on days 1-14 + etoposide 100 mg on days 1-5 + cisplatin 20 mg on days 2-5	etoposide 100 mg on days 1-5 + cisplatin 20 mg on days 2-5
Hu et al.2011	rh-Endostatin 15 mg on days 1-14 + topotecan 0.75-1.00 mg/m^2^ on days 1-5 + cisplatin 25 mg/m^2^; on days 1-3	topotecan 0.75-1.00 mg/m^2^ on days 1-5 + cisplatin 25 mg/m^2^; on days 1-3
Liu et al.2011	Thalidomide 100 mg/d daily + etoposide 100 mg/m^2^; on days 1-3 + cisplatin 30 mg/m^2^; on days 1-3	etoposide 100 mg/m^2^; on days 1-3 + cisplatin 30 mg/m^2^; on days 1-3
Cheng et al.2015	Thalidomide 150 mg/d daily + cisplatin 25 mg/m^2^ on days 1-3 + irinotecan 125 mg/m^2^ on days 1, 8	cisplatin 25 mg/m^2^ on days 1-3 + irinotecan 125 mg/m^2^ on days 1, 8
Liu et al.2015	Thalidomide 100 mg/d daily + etoposide 100 mg/m^2^; on days 1-3 + cisplatin 30 mg/m^2^; on days 1-3	etoposide 100 mg/m^2^; on days 1-3 + cisplatin 30 mg/m^2^; on days 1-3
Liu et al.2013	Thalidomide 100 mg/d daily + cisplatin 25 mg/m^2^ on days 1-3 + irinotecan 60 mg/m^2^ on days 1, 8	cisplatin 25 mg/m^2^ on days 1-3 + irinotecan 60 mg/m^2^ on days 1, 8
Pujol et al.2007	Thalidomide 100-400 mg/d + 4'-epidoxorubicin 40 mg/m^2^ on day 1 + cisplatin 100 mg/m^2^ on day 2 + etoposide 100 mg/m^2^ on days 1-3+ cyclophosphamide 400 mg/m^2^ on days 1-3 (every 28 days)	placebo 100-400 mg/d + 4'-epidoxorubicin 40 mg/m^2^ on day 1 + cisplatin 100 mg/m^2^ on day 2 + etoposide 100 mg/m^2^ on days 1-3+ cyclophosphamide 400 mg/m^2^ on days 1-3 (every 28 days)
Lee et al.2009	Thalidomide 100-200 mg/d + etoposide 120 mg/m^2^ on day 1 and 200 mg on days 2-3 + carboplatin AUC = 5 mg/ml/min (extensive-stage) or AUC = 6 mg/ml/min (limited-stage) on day 1	placebo 100-200 mg/d + etoposide 120 mg/m^2^ on day 1 and 200 mg on days 2-3 + carboplatin AUC = 5 mg/ml/min (extensive-stage) or AUC = 6 mg/ml/min (limited-stage) on day 1
Arnold et al.2007	Vandetanib 300 mg/d daily	placebo 300 mg/d daily

Abbreviations: ACT: angiogenesis inhibitors plus chemotherapy; CT: chemotherapy; AUC: area under the curve.

### Methodological quality

**Figure 2:**
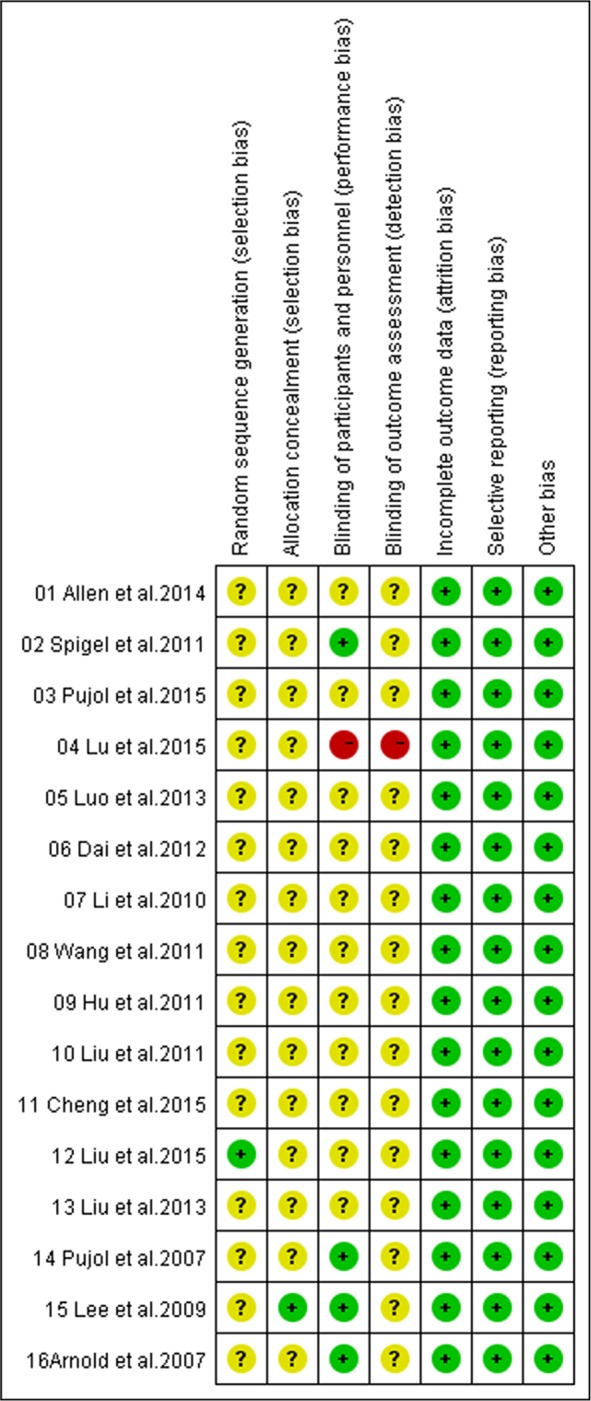
Bias risk and quality assessment of included studies

In accordance with the recommendations of the Cochrane Handbook for Systematic Reviews, we assessed the eligible trials using the seven aspects mentioned above. Among these 16 recruited trials, all referred to the use of random allocation, and one [27] of them discussed the methods, four [17, 29–31] performed or reported their blinding methods and one [30] reported its allocation concealment. All trials applied the intent-to-treat analysis and underwent quality assessment. Eventually, fifteen [16–18, 20–31] received B quality scores and one [19] received C quality score (Figure 2).

### Objective response rate

**Figure 3:**
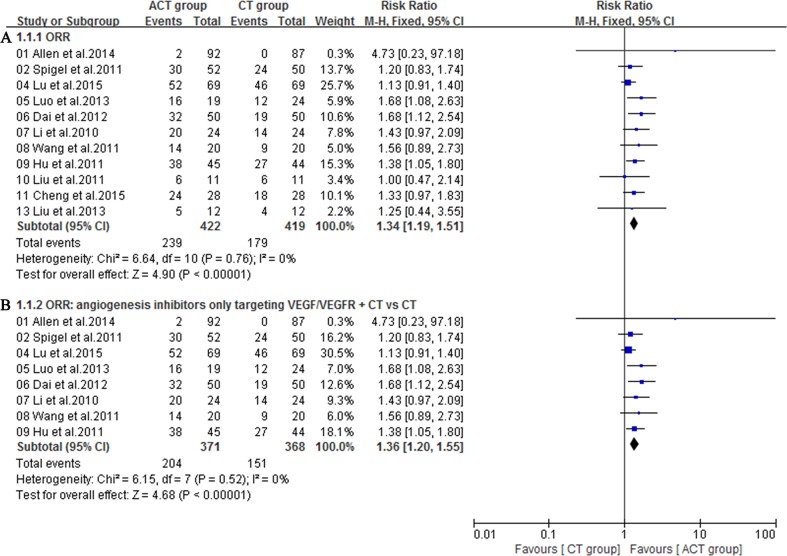
**A.** Objective response rate (ORR) of the studies; **B.** Subgroup analysis of ORR for angiogenesis inhibitors only targeting VEGF/VEGFR plus chemotherapy (CT) versus CT.

**Figure 4:**
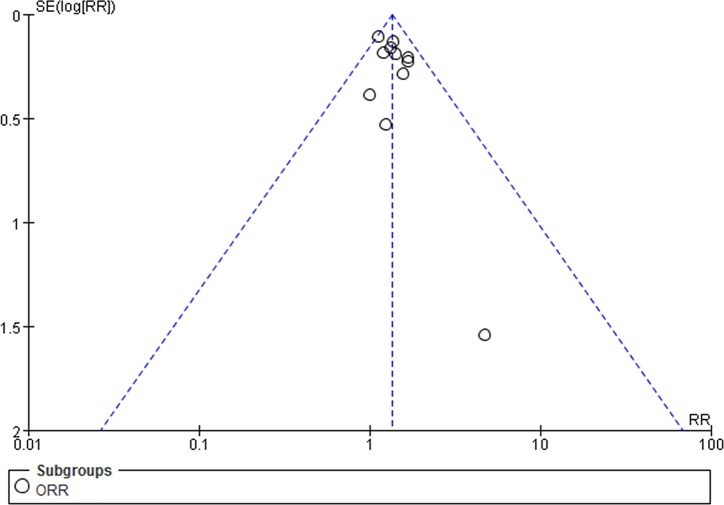
Funnel plot of ORR for included studies

Eleven eligible trials [16, 17, 19–26, 28] involved objective response rate (ORR), which was defined as the proportion of complete and partial responses among all evaluable patients. A fixed-effect model was utilized because heterogeneity did not exist (*I*^2^ = 0%). Notably, angiogenesis inhibitors plus chemotherapy group exhibited a superior ORR to chemotherapy alone group (RR = 1.34; 95% CI = 1.19-1.51; *P <*0.00001) (Figure 3). The funnel plot indicated no significant publication bias on ORR (Figure 4). Additionally, subgroup analysis on angiogenesis inhibitors only targeting VEGF/VEGFR (Bevacizumab, Ziv-aflibercept, rh-Endostatin) also acquired a superior ORR (RR = 1.36; 95% CI = 1.20-1.55; *P* <0.00001) (Figure 3).

### Survival

**Figure 5:**
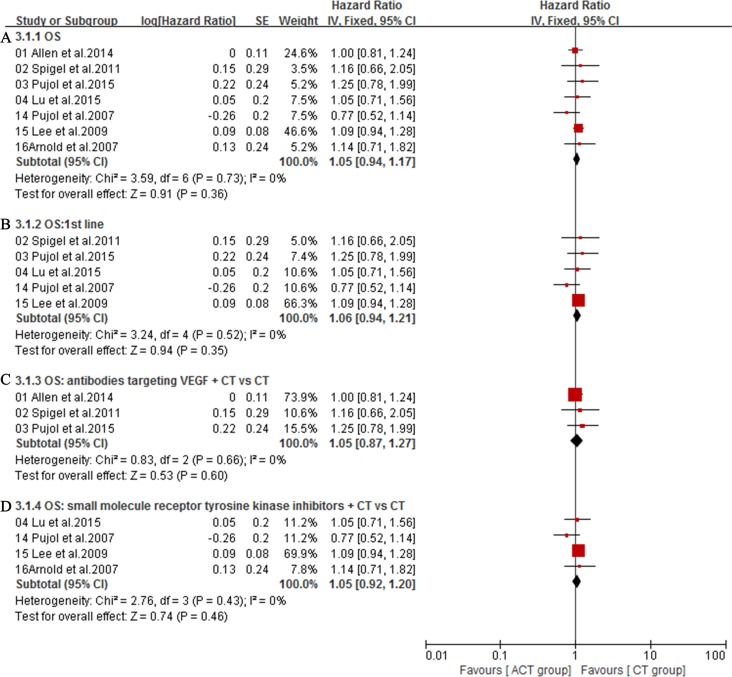
**A.** Overall survival (OS) of the studies; **B.** Subgroup analysis of the effect of angiogenesis inhibitors on OS in first-line settings; **C.** Subgroup analysis of OS for antibodies targeting VEGF plus CT versus CT; **D.** Subgroup analysis of OS for small molecule angiogenesis inhibitors plus CT versus CT.

**Figure 6:**
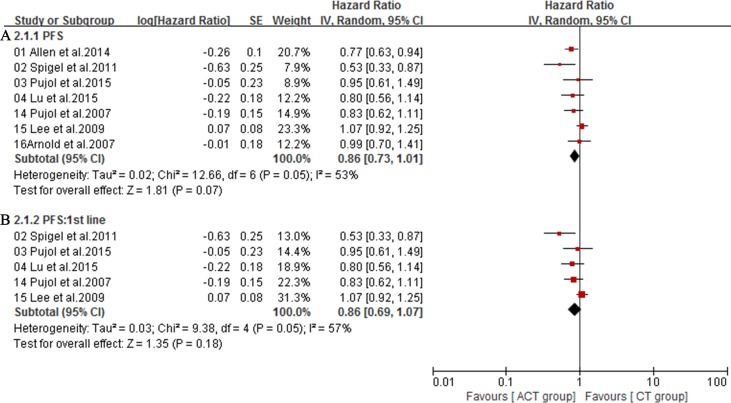
**A.** Progression-free survival (PFS) of the studies; **B.** Subgroup analysis of the effect of angiogenesis inhibitors on PFS in first-line settings.

**Figure 7:**
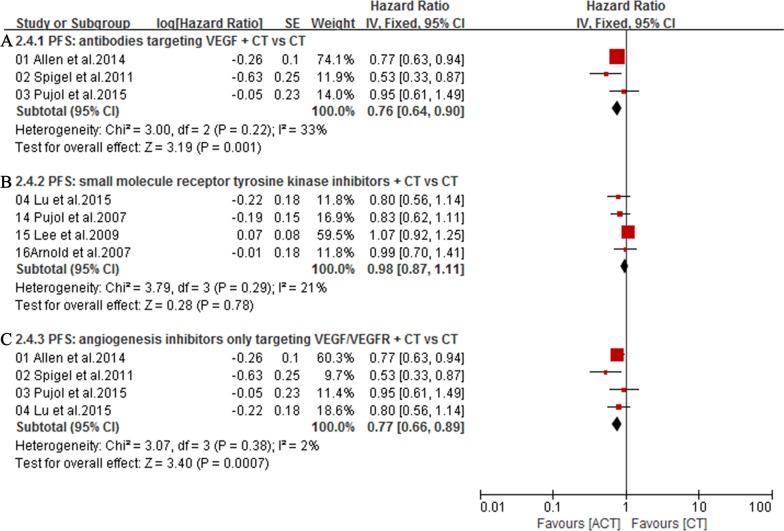
**A.** Subgroup analysis of PFS for antibodies targeting VEGF plus CT versus CT; **B.** Subgroup analysis of PFS for small molecule angiogenesis inhibitors plus CT versus CT; **C.** Subgroup analysis of PFS for angiogenesis inhibitors only targeting VEGF/VEGFR plus CT versus CT.

The overall survival (OS) was available in 7 trials [16–19, 29–31]. Owing to the heterogeneity values (*I*^2^ = 0%), a fixed-effect model was employed and the result showed no significant difference in OS between ACT group and CT group (HR = 1.05; 95% CI = 0.94-1.17; *P =* 0.36) (Figure 5). Additionally, subgroup analysis showed that, compared with chemotherapy alone group, first-line treatment with angiogenesis inhibitors plus chemotherapy did not significantly lower mortality risk (HR = 1.06; 95% CI = 0.94-1.21; *P* = 0.35) (Figure 5). Meanwhile, compared with chemotherapy alone group, antibodies targeting VEGF (HR = 1.05; 95% CI = 0.87-1.27; *P* = 0.60) (Figure 5) or small molecule angiogenesis inhibitors (HR = 1.05; 95% CI = 0.92-1.20; *P* = 0.46) (Figure 5) plus chemotherapy group also did not significantly lower mortality risk. Seven trials [16–19, 29–31] reporting PFS were analyzed via a random-effects model based on the heterogeneity values (*I*^2^ = 53%) and revealed that, compared with chemotherapy alone, angiogenesis inhibitors plus chemotherapy slightly prolonged PFS (HR = 0.86; 95% CI = 0.73-1.01; *P =* 0.07) (Figure 6). Angiogenesis inhibitors in first-line setting had no benefits in PFS (HR = 0.86; 95% CI = 0.69-1.07; *P* = 0.18) (Figure 6). Subgroup analysis showed that, compared with chemotherapy alone, the addition of antibodies targeting VEGF significantly prolonged PFS (HR = 0.76; 95% CI = 0.64-0.90; *P* = 0.001) (Figure 7) while the addition of small molecular receptor tyrosine kinase inhibitors yielded no benefits in PFS (HR = 0.98; 95% CI = 0.87-1.11; *P* = 0.78) (Figure 7). Additionally, subgroup analysis on angiogenesis inhibitors only targeting VEGF/VEGFR (Bevacizumab, Ziv-aflibercept, rh-Endostatin) also acquired a superior PFS (HR = 0.77; 95% CI = 0.66-0.89; *P* = 0.0007) (Figure 7).

### Adverse events

**Figure 8:**
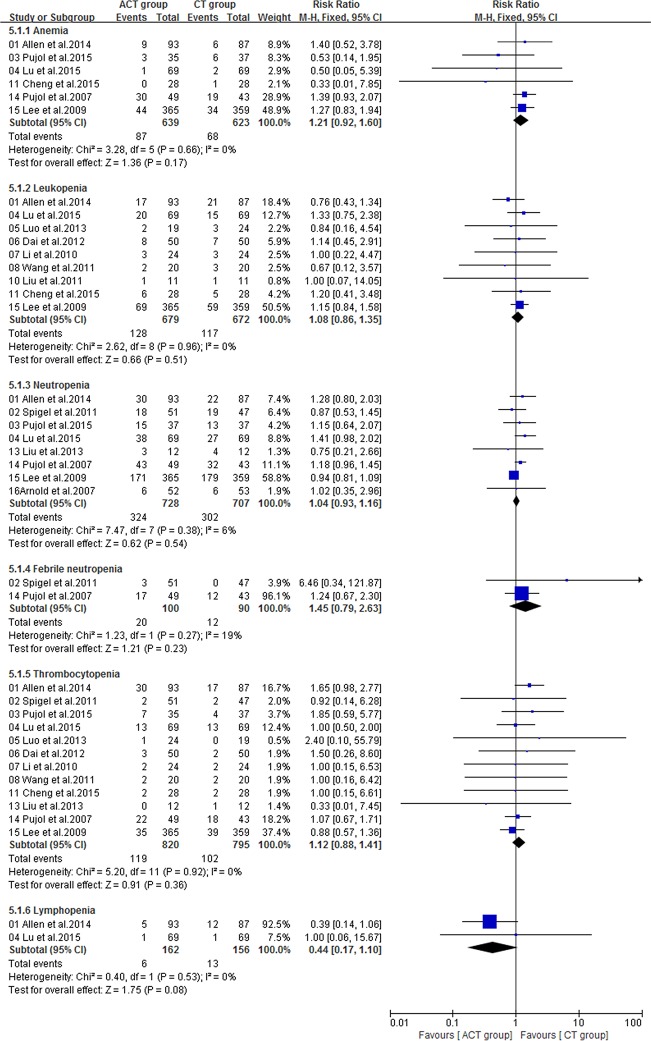
Severe hematologic toxicities of the studies

**Figure 9:**
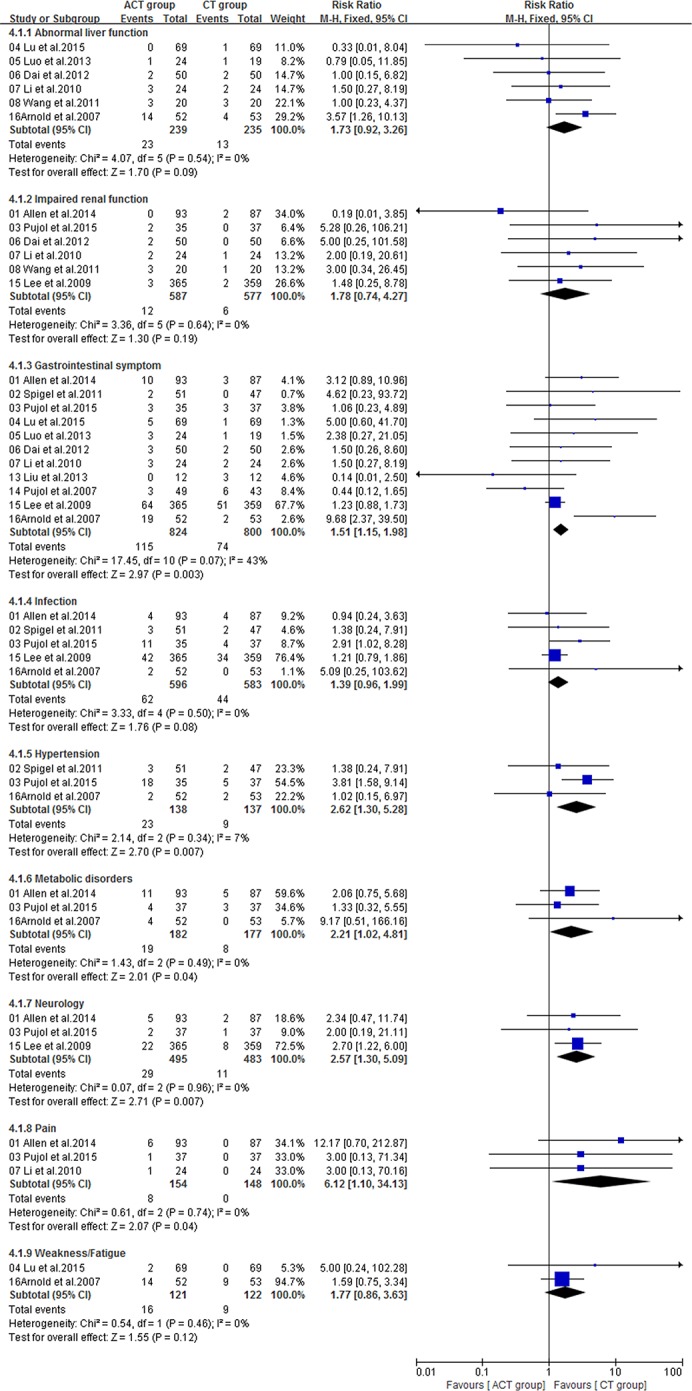
Severe nonhematologic toxicities of the studies (Part 1)

**Figure 10:**
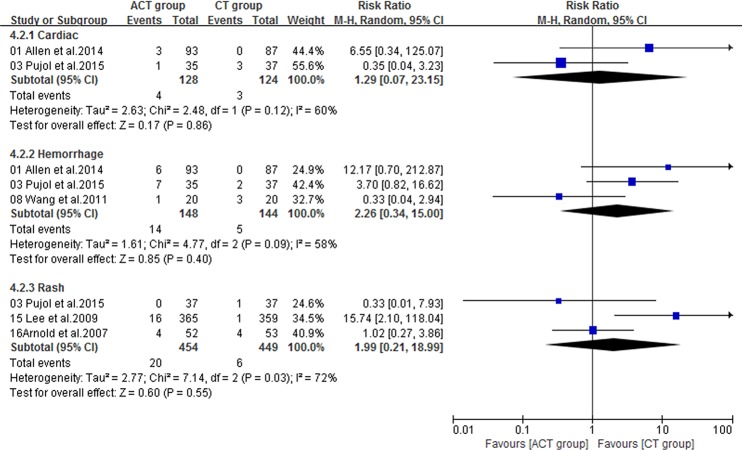
Severe nonhematologic toxicities of the studies (Part 2)

Fourteen included trials [16–23, 25, 26, 28–31] with sufficient data of treatment-related toxicity and severe AEs grading were applied to analyze AEs (Grade≥ 3). Severe hematotoxicity was the most common AEs without a significant difference between ACT and CT group (Figure 8). On the other hand, the most common non-hematologic AEs were largely mild and tolerable without a significant difference between two arms, with the exception that more patients in ACT group had gastrointestinal symptom (RR = 1.51; 95% CI = 1.15-1.98; *P* = 0.003), hypertension (RR = 2.62; 95% CI = 1.30-5.28; *P* = 0.007), metabolic disorders (RR = 2.21; 95% CI = 1.02-4.81; *P* = 0.04), neurology (RR = 2.57; 95% CI = 1.30-5.09; *P* = 0.007) or pain (RR = 6.12; 95% CI = 1.10-34.13; *P* = 0.04) (Figure 9, 10).

## DISCUSSION

To our best knowledge, this study is the only meta-analysis to investigate antiangiogenic therapy in SCLC. Antiangiogenic agents consist mainly of antibodies (bevacizumab, ziv-aflibercept) and small molecules agents (rh-endostatin, vandetanib and thalidomide). The antibodies ‘deactivate’ VEGF via binding to them and blocking their bond to relevant receptors [32], while small molecules agents directly inhibit the kinase activity of VEGFR by competitively binding to receptor tyrosine kinases [33]. This study showed that the addition of angiogenesis inhibitors to chemotherapy possessed a significant benefit in terms of ORR, slightly prolonged the median PFS. Indeed, some small molecules agents analyzed in this study were mainly multikinase-inhibitors (e.g. vandetanib: EGFR VEGFR RET) or substances (e.g. thalidomide) which affected a lot of cellular processes and antiangiogenesis was only part of anti-cancer mechanisms [34–37]. Thus the benefits of ORR and PFS could not be clearly attributed to anti-angiogenetic effect. Therefore, we conducted subgroup analysis on angiogenesis inhibitors only targeting VEGF/VEGFR (Bevacizumab, Ziv-aflibercept, rh-Endostatin). The results showed that anti-angiogenetic effect played a critical role in improving ORR and PFS. However, the improvement of ORR and PFS failed to translate into an OS benefit. One possible explanation was that the activation of alternative pro-angiogenic factors swiftly counteracted the effect of angiogenesis inhibitors; another was intrinsic or pre-existing resistance [38]. Considering the fundamental distinction between small molecule angiogenesis inhibitors and antibodies targeting VEGF in many aspects, we further conducted a subgroup analysis of both groups. The results showed that, compared with chemotherapy alone, neither antibodies targeting VEGF nor small molecule angiogenesis inhibitors plus chemotherapy significantly lowered mortality risk. Encouragingly, subgroup analysis exhibited that antibodies targeting VEGF plus chemotherapy significantly prolonged PFS for patients with SCLC. A reasonable explanation was that chemotherapy failed to inhibit angiogenesis in the presence of a reactive resistance, which was mediated by the HIF-1/VEGF pathway in cancer cells, while antibodies targeting VEGF blocked the reactive resistance, sensitizing cancer cells to chemotherapy [39].

VEGF is also a key mediator of angiogenesis in healthy tissues. VEGF may induce vasodilation by stimulating the release of nitric oxide in endothelial cells [40]. Therefore, inhibition of VEGF may lead to vasoconstriction and result in an increased peripheral vascular resistance [41]. Consequently, the use of angiogenesis inhibitors can lead to vascular disturbances which are the main factor behind the AEs of these drugs [42]. Our study showed that the addition of angiogenesis inhibitors would increase some common adverse events, such as pain, gastrointestinal symptom, hypertension, metabolic disorders and neurology.

Nevertheless, this study confronted following limitations: (i) eligible trials adopted several kinds of antiangiogenic agents; (ii) clinical characteristics such as ECOG performance status as well as stage were not completely equivalent; (iii) trials were mainly conducted in a molecularly unselected population. Thus, it impeded us to conduct a sub-analysis of potential predictive biomarkers to identify the exact benefit population.

Briefly, compared with chemotherapy alone, antibodies targeting VEGF plus chemotherapy significantly improved ORR and prolonged PFS with an acceptable toxicity profile for patients with SCLC. Therefore, angiogenesis inhibitors, especially antibodies targeting VEGF, combining with chemotherapy may be a potential promising strategy in managing SCLC.

## METHODS

### Data sources and search

Two authors (Lin H and Li LN) independently carried out a comprehensive systematic search for published articles in PubMed, EMBASE, Cochrane Library and Wanfang Data (a literature search database from China) without language restriction from inception to March 2016, in accordance with PRISMA (Preferred Reporting Items for Systematic Reviews and Meta-Analyses) guidelines [43], using following keywords and Mesh terms: “(“angiogenesis inhibitors” [all fields] OR “antiangiogenesis” [all fields] OR “antiangiogenic agents” [all fields]) AND (“ziv-aflibercept” [all fields]) AND (“bevacizumab” [all fields]) AND (“rh-Endostatin” [all fields]) AND (“thalidomide” [all fields]) AND (“vandetanib” [all fields]) AND (“chemotherapy” [all fields]) AND (“small cell lung cancer” [Mesh] OR “SCLC” [Mesh])”. Reviews, preclinical and animal trials were excluded.

### Study Selection

The inclusion criteria were as follows: (i) RCT or clinical controlled trails with voluntarily enrolled patients; (ii) all participants had been histologically or cytologically confirmed; (iii) the trials involving angiogenesis inhibitors plus chemotherapy versus chemotherapy alone; (iv) trials excluded patients with double or multiple primary cancer or presence of unstable systemic disease; (v) trials evaluated at least one of ORR, OS, PFS and severe AEs (Grade≥3); (vi) response rate was determined using the Response Evaluation Criteria in Solid Tumors (RECIST 1.0 or 1.1 standards) [44] or WHO criteria [45]; (vii) adverse events were evaluated according to the National Cancer Institute Common Terminology Criteria for Adverse Events (CTCAE 2.0 or 3.0) [46] or WHO criteria [45].

### Data extraction and quality assessment

Data were extracted independently by two reviewers (Lin H and Li LN), and any disagreements between the two reviewers were resolved by consensus involving a third reviewer (Xie XH). For each selected publication, we extracted the following items: first author, year of publication, country of original trial, trial phase, line of treatment, number of patients, demographics, clinical parameters, stage at initial diagnosis, interventions and outcomes. To assess study quality and applicability, we used the checklists of The Cochrane Handbook for Systematic Reviews of intervention (Version 5.1.0), based on the following criteria: (i) Random sequence generation; (ii) Allocation concealment; (iii) Blinding of participants and personnel; (iv) Blinding of outcome assessment; (v) Incomplete outcome data; (vi) Selective reporting; (vii) Other bias. Each trial for bias based on the criteria listed above was marked as ‘low risk’, ‘high risk’ or ‘unclear risk’. Trials quality was defined as following: A rating: meeting all criteria of low risk; B rating: meeting one or more criteria of unclear risk without high risk; C rating: appearing one or more criteria of high risk.

### Statistical analysis

Statistical analysis was performed using RevMan 5.3 software (Cochrane Collaboration's Information Management System). Analysis of data comprised pooled risk ratio (RR) for dichotomous endpoints (e.g ORR, severe AEs), using the Mantel-Haenszel method [47]. The events and total number of patients from ACT group and CT group in the trials for ORR and severe AEs were extracted from the trials. OS and PFS were calculated using effect variables and expressed by hazard ratio (HR). HRs with 95% confidence intervals (CI) were directly extracted from trials or from the survival curves using the methods described by Tierney et al. [48] for OS and PFS when HRs were unavailable. The 95% CIs were calculated and presented in forest plots. Two-sided *P* values less than 0.05 were considered statistically significant. Statistical heterogeneity of different trials was evaluated with the *I*-square tests [49]; no heterogeneity existed when *I*^2^ < 50%, a fixed-effect model was applied to pool the study results. Significant heterogeneity was found if *I*^2^ > 50%, and a random-effects statistical model was used [50]. The risk of publication bias was evaluated via visual appraisal of funnel plots.
